# Deep tissue photoacoustic imaging of nickel(II) dithiolene-containing polymeric nanoparticles in the second near-infrared window

**DOI:** 10.7150/thno.39403

**Published:** 2020-01-22

**Authors:** Byullee Park, Kyung Min Lee, Suhyeon Park, Misun Yun, Hak-Jong Choi, Jeesu Kim, Changho Lee, Hyungwoo Kim, Chulhong Kim

**Affiliations:** 1 Departments of Creative IT Engineering, Electrical Engineering, and Mechanical Engineering, Pohang University of Science and Technology (POSTECH), 77 Cheongam-ro, Pohang 37673, Republic of Korea.; 2 Department of Materials Science and Engineering, College of Engineering, Seoul National University, Seoul 08826, Republic of Korea.; 3 Interdisciplinary Program of Molecular Medicine, Chonnam National University, 77 Yongbong‐ro, Buk‐gu, Gwangju 61186, Republic of Korea.; 4 Microbiology and Functionality Research Group, World Institute of Kimchi, 86 Kimchi-ro, Gwangju 61755, Republic of Korea.; 5 Department of Nuclear Medicine, Chonnam National University Medical School & Hwasun Hospital, 264, Seoyang-ro, Hwasun-eup, Hwasun-gun, Jeollanam-do 58128, Republic of Korea.; 6 School of Polymer Science and Engineering, Chonnam National University, 77 Yongbong-ro, Buk-gu, Gwangju 61186, Republic of Korea.

**Keywords:** Photoacoustic imaging, Deep tissue imaging, Nickel dithiolene complex, Polymeric nanoparticle, Second near-infrared window.

## Abstract

Photoacoustic imaging is gaining great attention in the medical world due to its significant potential for clinical translation. Light excitation in the second near-infrared (NIR-II) window (1000-1350 nm) has resolution and penetration depth suitable for several clinical applications. However, the significant challenge exists for clinical translation because of the absence of notable intrinsic chromophores in this clinically significant optical range to generate diagnostic images.

**Methods**: We present newly developed a biocompatible nickel dithiolene-based polymeric nanoparticle (NiPNP), which have a strong and sharp absorption peak at 1064 nm, as a photoacoustic contrast agent to boost specific absorbance in the NIR-II window for *in vivo* deep tissue imaging.

**Results**: We confirm the enhanced PA signal by NiPNP's strong light absorption in the NIR-II window (287% higher than that of NIR-I) and deep tissue imaging capability (~5.1 cm) through *in vitro* experiment. We have successfully acquired diagnostic-quality *in vivo* photoacoustic images in deep tissue (~3.4 cm) of sentinel lymph nodes, gastrointestinal tracts, and bladders of live rats by using clinically viable imaging system.

**Conclusions**: Our results prove that with strong absorption in the NIR-II window and with deeper imaging depth, the clinical translation of photoacoustic imaging with NiPNP is feasible for preclinical studies and thus would facilitate further clinical investigations.

## Introduction

Photoacoustic imaging (PAI) is a rapidly growing non-invasive imaging modality that has proven its feasibility in preclinical and clinical investigation 1-7. The PAI provides structural and functional information of live animals and human tissues based on the optical absorption by intrinsic chromophores such as hemoglobin, melanin, and lipids 8-12. However, PAI using endogenous chromophores alone has difficulties in imaging some specific organs such as lymph nodes, gastrointestinal (GI) tracts, and bladders due to their optical transparency. Consequently, it is difficult to achieve high sensitivity and specificity to diagnose diseases related to these specific organs. In addition, optical absorption by unwanted intrinsic chromophore present within the tissue generates background noise, resulting in low image contrast in PAI 13-16. To overcome these obvious disadvantages of intrinsic molecular PAI, it is essential to design exogenous agents with strong light absorption properties at the appropriate optical wavelength to generate high quality diagnostic PA images.

Various studies using exogenous contrast agents and near-infrared (NIR) light have been conducted to overcome the limitations of intrinsic chromophores in PAI 17-25. In particular, the low attenuation of NIR light compared to the visible light in the tissue is advantageous for acquiring deep tissue PAI *in vivo* 26, 27. Many studies were performed with methylene blue, indocyanine green, and various nanoparticles as the exogenous agent in the first NIR window (NIR-I, 650-950 nm) 28-34. Recently, PAI using the second NIR window (NIR-II, 1000-1650 nm) light has received much attention than with the NIR-I because of the following reasons: (1) low photon energy at the longer wavelengths is more suitable for preclinical and clinical studies since they reduce tissue damages; (2) the NIR-II light gets less scattered in biological tissues and avoids absorption by the intrinsic chromophores to minimize background noise and maximize penetration depth; (3) compared to lasers available in the NIR-I window, the 1064-nm Nd:YAG lasers are inexpensive, widely available, and more compact possibly leading to a commercial PAI system beyond research. In addition, because the 1064-nm Nd:YAG lasers have already been used widely in clinical areas such as dermatology, it is well suited for translational research 35-46. The main challenges for performing PAI in the NIR-II window and applying to preclinical and clinical fields hinge on the design of PA contrast agents that can effectively absorb the NIR-II light and their feasibility of *in vivo* PAI 47-51. Several studies have been reported in the literature on NIR-II light absorption agents, e.g., gold nanorods 36, 44, narrow-bandgap polymer nanoparticles 40, 41, 43, phosphorus phthalocyanines 39, copper sulfide nanoparticles 37, silver nanoplates 35, 42, perfluorocarbon nanodroplet 38. Even though the agents above confirmed the possibility of using a 1064-nm laser as an excitation source for PAI, most *in vivo* studies could be performed only at shallow depths 35, 37, 39-41, 43, 44. Moreover, some agents showed their absorbance positioned predominantly in NIR-I region rather than NIR-II (inefficient absorption in NIR-II window) 37, 42 and were not even feasible for *in vivo* PAI 36, 38. Recently, a cyanine-based agent was reported as a form of surfactant-stripped micelle and provided notable deep-tissue imaging in NIR-II window 52. However, it is a still rare case of using small-molecule dyes so far, and other various NIR-II absorbing organic dyes are worth further investigation 53.

In this study, we are the first to report a nickel dithiolene-based polymeric nanoparticle (NiPNP) as a PAI contrast agent that strongly absorbs the NIR-II light with a peak at 1064 nm for *in vivo* deep tissue PAI. Many dithiolene complexes have been investigated widely for non-linear optics, telecommunication, thermoelectrics, or catalytic hydrogen evolution due to unique optical and electronic properties as well as redox and catalytic activities 54. In particular, nickel dithiolene complexes have attracted attention because of thermo- and photostability and their distinctive photophysical properties including broad NIR absorbance window, which can be further tailored by altering chemical structure of peripheral ligands 55-59. However, the limited solubility of the complex reduces processability and restricts other potential applications. We used bis(4-dimethylaminodithiobenzil)nickel(II) for deep tissue PAI, and prepared nanosized particles (~130 nm) benefitted from an FDA-approved poly(lactide-*co*-glycolide) (PLGA) via nanoprecipitation 60. The core complex was reported show a high molar extinction coefficient (*ε,* 2.8 × 10^4^ M/cm at 1060 nm) in the NIR region 57 and also high photochemical stability when used in nonlinear optics or optoelectronics 56, 61, 62, which are sought-after, essential properties for the NIR bio-imaging. Therefore, we could demonstrate the facile preparation of the strong NIR-II absorbing and biocompatible PAI agent that is comprised of the photoactive core and the PLGA shell. The NiPNP disperses stably in an aqueous medium and exhibits strong NIR absorption between 800-1300 nm with a peak at 1064 nm attributed to the electron transition in the nickel complex and is most suitable for NIR-II PA imaging in deep tissues.

## Materials and Methods

### Materials

Bis(4-dimethylaminodithiobenzil)nickel(II) was purchased from TCI (Tokyo, Japan) and poly(lactic-*co*-glycolic acid) (PLGA; Resomer^®^ RG 503H) from Aldrich. The PLGA consists of 50:50 lactic acid-glycolic acid with a weighted average molecular weight (M_w_) ranging from 24 to 38 kDa. All reagents used were purchased commercially and were used as is unless otherwise noted.

### Optical and physical characterization

Transmission electron microscopy (TEM) image was obtained using by a JEM-3010 (JEOL, JPN) operating at 300 kV. The sample solution was dropped on a carbon-coated copper grid and dried under vacuum at room temperature for 24 h. The analytical work was performed by energy-dispersive X-ray spectroscopy (EDS, Oxford Instruments, UK), equipped in TEM. Hydrodynamic particle size and zeta potential were recorded using an electrophoretic light scattering analyzer (ELS-8000, Otsuka Electronics). Optical absorption spectra were measured using a UV-Vis-NIR spectrophotometer (V-770, JASCO). Fourier-transform infrared spectroscopy (FT-IR) were performed using an Attenuated total reflectance Fourier-transform infrared spectroscopy (ATR-FTIR, IFS66/S, Bruker, USA).

### Synthesis of NiPNP

NiPNP was synthesized by a typical nanoprecipitation method with minor modification. Bis(4-dimethylaminodithiobenzil)nickel(II) (80 mg, 0.127 mmol) was added to a solution of poly(lactic-*co*-glycolic acid) (PLGA; 267 mg) in acetone (80 mL). After a brief bath sonication, the resulting solution was introduced dropwise to the deionized (DI) water (200 mL) for 10 min while stirring vigorously. The mixture was further stirred for 24 h in a fume hood at 60 °C to remove the acetone and filtered using a 0.2-µm syringe filter to afford a colloid of NiPNP (17 mg/mL). The concentration of dispersion was adjusted by adding DI water for dilution, or by evaporating under reduced pressure and re-adding desired amounts of DI water for higher concentrations.

### *In vitro* toxicity study

The mouse fibroblast L929 cell was purchased from the Korean cell line bank, and MTT kit and live/dead assay were performed to analyze the L929 cell growth under treatment with NiPNP. The cells were harvested and counted, and seeded into 96-well plates at the density of 5,000 viable cells per well and then incubated overnight. 48 hrs after treatment, MTT kit solution was added to the cells and incubated for 24 hrs at 37 ºC. The amount of formazan produced was measured at 562 nm wavelength using an infinite M200 PRO microplate reader. Under same experimental condition, we conducted live/dead assay after incubating NiPNP treated cells during 30 minutes at 20-25^o^C and took florescent microscopic images.

### *In vivo* toxicity study

Six white Balb/c mice were prepared for *in vivo* toxicity studies by intravenously injecting NiPNP with a concentration of 17.3 mg/mL of 100 μL. Three mice with PBS intravenous injection were used for the control group. 7 and 28 days after the injection of NiPNP, histological and blood analyses were performed on critical organs (i.e., liver, lung, kidney, spleen, heart). Histological data of these organs were fabricated by following the conventional H&E staining process. The blood biochemistry and complete blood cell count were measured.

### ICP-MS experiments of extracted organs

NiPNP and PBS were intravenously injected (100 mg/kg) into 6-week female normal Balb/c mice *(n=5 for NiPNP and n=3 for PBS)*. After 24 hours, a mouse was completely anesthetized via a vaporized-isoflurane kit and sacrificed by cervical dislocation. Then, the liver, kidneys, spleen, heart and lungs were extracted. After weighting these organs, they were fully dissolved via fresh aqua regia (5 mL) and heated at 130 ^o^C. By diluting this solution with DI water, ICP-MS (NexION 350D, Perkin-Elmer SCIEX) calculated the concentration of Ni.

### Photoacoustic imaging system

We used two PAI systems: one for *in vitro* and the other for *in vivo and ex vivo* experiments. First, for *in vitro* imaging, an acoustic-resolution photoacoustic microscopy (AR-PAM) system which was introduced in our previous study 10 was used to analyze the photoacoustic (PA) characteristics of NiPNP (Figure S1). Second, for *in vivo and ex vivo* PA and ultrasound (US) imaging, a clinical PA/US system was used to acquire the data. The clinical PA/US imaging system (Figure S2) was divided into an FDA approved programmable US imaging system (EC-12R, Alpinion Medical Systems) and a portable laser system (Phocus, OPOTEK). The laser consisted of an Nd: YAG pumping source and a tunable OPO, which allowed selective use of wavelengths of 532, 1064 and 680 - 900 nm as a PA excitation source. The laser illumination of the selected wavelength was coupled with the bifurcated fiber bundles connected to the laser body. The US imaging system connected to a transducer (L3-12, Alpinion Medical Systems, 8.5 MHz center frequency with a fractional bandwidth of 95%) transmitted and received US signals to form US image and also received the PA signals generated by the laser to form PA image. The US imaging system was synchronized with the laser system by the trigger signal that the laser system sent every pulse. The US transducer and the bifurcated fiber bundle were inserted into the adapter, fixed with forceps, and connected to a single axis motor. For the volumetric *in vivo* PA/US imaging, Y direction raster scanning was performed by the single axis motor.

### *In vitro*, *in vivo*, and *ex vivo* PA experiments

The AR-PAM system was used for *in vitro* PA experiments. Eight tubes (508-001, Silastic Laboratory Tubing, Dow Corning, USA), similar in size to the blood vessel, were filled with NiPNP having a concentration of 34.6, 17.3, 11.5, 6.8, 3.4, 1.7, 0.8 and 0 mg/mL. The PA spectra were measured from 680 to 1064 nm. For the NiPNP with a concentration of 17.3 mg/mL, additional PA spectra from 1200 to 1350 were also measured. The laser power for each wavelength was measured using a power meter (S350C, Thorlabs, USA) and the power was adjusted to the same value by adjusting the Q-delay of the laser. For the *in vitro* PAI of NiPNP in deep chicken breast tissues, the laser of the AR-PAM system and the programmable US imaging system were used together. The 1064 nm laser fluence used in the experiment was 40 mJ/cm^2^, well below the American National Standards Institute (ANSI) laser safety limit of 100 mJ/cm^2^. For the sample preparation, a 1 mL of NiPNP with a concentration of 17.3 mg/mL was injected into the microtube, and the microtube was sealed with a silicone mixture to prevent bubble formation. Five layers of 1-cm-thick chicken breasts tissue were stacked on prepared tubes (Figure S3), and two-dimensional depth-resolved PA images were obtained at each layer. To improve the signal-to-noise ratio (SNR) of the PA image, a total of 100 images were collected at the same spatial location and averaged.

The clinical PA/US imaging system was used for *in vivo* sentinel lymph nodes (SLNs), GI tracts, and bladders PA imaging of rats. Pulsed laser at a 1064 nm (30 mJ/cm^2^) was used for *in vivo* experiments. All animal experimental procedures were performed in accordance with laboratory animal use protocol approved by the institutional animal care and use committee of the Pohang University of Science and Technology. Healthy rats (~200 g) were prepared to acquire PA/US images. A vaporized-isoflurane system (1 L/min of oxygen and 0.75% isoflurane) was used to initially anesthetize rats and maintain anesthesia during the experiment. After acquiring control PA images for SLN, GI tract, and bladder, NiPNP was delivered through hypodermic injection, oral administration, and transurethral injection (0.1, 0.3, and 0.2 mL of 17.3 mg/mL, respectively) into three different targets, respectively. After a certain time lapse, PA images were obtained at the same location as the control PA images. In the case of PA SLNs, *ex vivo* PA images were obtained by dissecting NiPNP-containing lymph nodes and normal lymph nodes, to validate the *in vivo* results.

## Results

### Synthesis of Ni(II)-containing polymeric nanoparticles and their optical and physical properties

**Figure 1:**
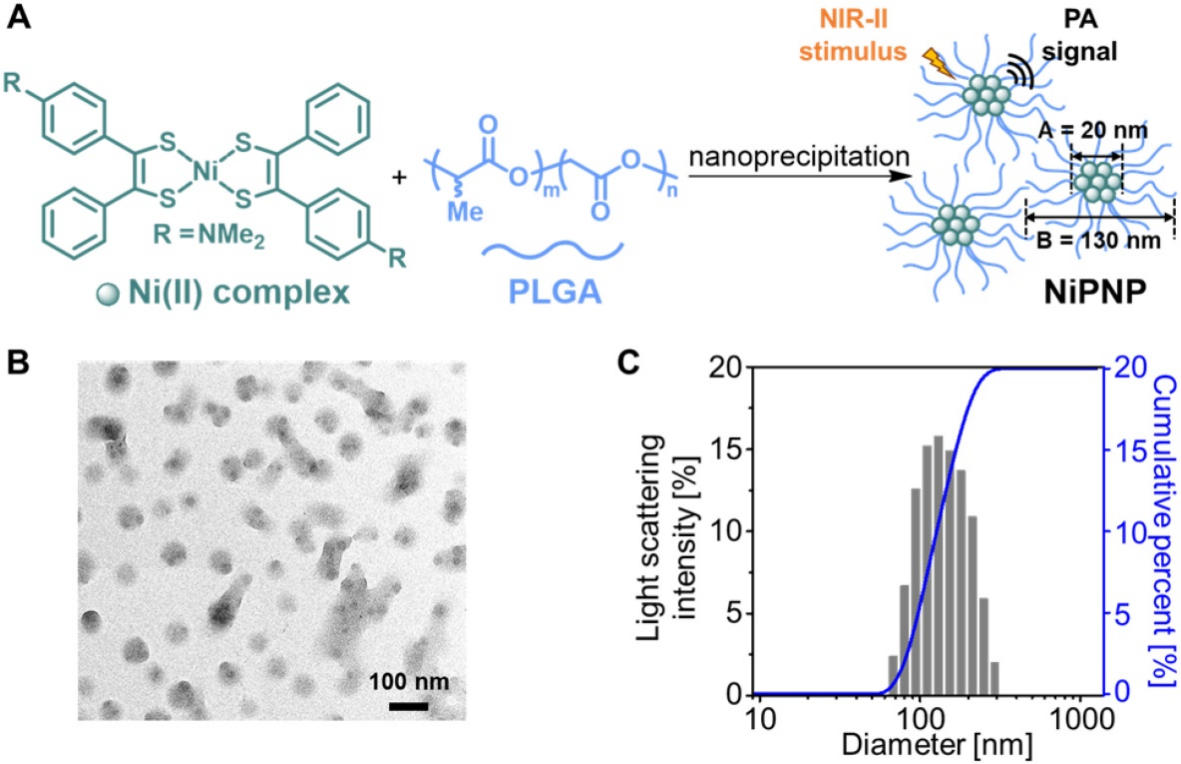
(A) Depiction for the preparation of NiPNP. The core Ni(II) complex (A; diameter, 20 nm) was encapsulated with PLGA via nanoprecipitation to form NiPNP (B; hydrodynamic size, 130 nm). (B) TEM image of NiPNP. (C) Particle size distribution of NiPNP obtained using DLS analysis. NiPNP, Ni(II) complex-containing polymeric nanoparticles; PLGA, poly(lactic-co-glycolic acid); TEM, transmission electron microscope; DLS, dynamic light scattering; NIR, near-infrared.

A one-step nanoprecipitation method was used to synthesize the core-shell type NiPNP. The NiPNP contains bis(4-dimethylaminodithiobenzil)nickel(II) (BDN) as a PA-active core material and PLGA as a biocompatible polymer shell, as shown in Figure 1A. The core Ni(II) complex exhibits broad light absorption in the NIR-II window and allows the use of laser pulse at 1064 nm while PLGA encapsulates the core complexes, which significantly increases the penetration depth for deep tissue imaging. Therefore, after precipitation process, the nanoparticle agent was obtained which showed the desired absorption spectrum in the NIR-II window in water. Figure 1B shows the core-shell structure of NiPNPs in which the size of the photoactive BDN core in NiPNPs measured an average of 20 ± 2.6 nm when observed by TEM. The EDS equipped in TEM confirmed the chemical composition of an individual NiPNP particle including elemental Ni(II) (Figure S4). We also investigated a hydrodynamic diameter of the nanoparticles including the polymer shell through dynamic light scattering measurement which reflects a fully extended, overall size of particle under aqueous conditions, and found the value of 130 ± 52 nm with a uniform distribution (polydispersity index of 0.10) (Figure 1C). FT-IR investigation further revealed the inclusion of the metal complex and PLGA to afford NiPNP (Figure S5).

**Figure 2:**
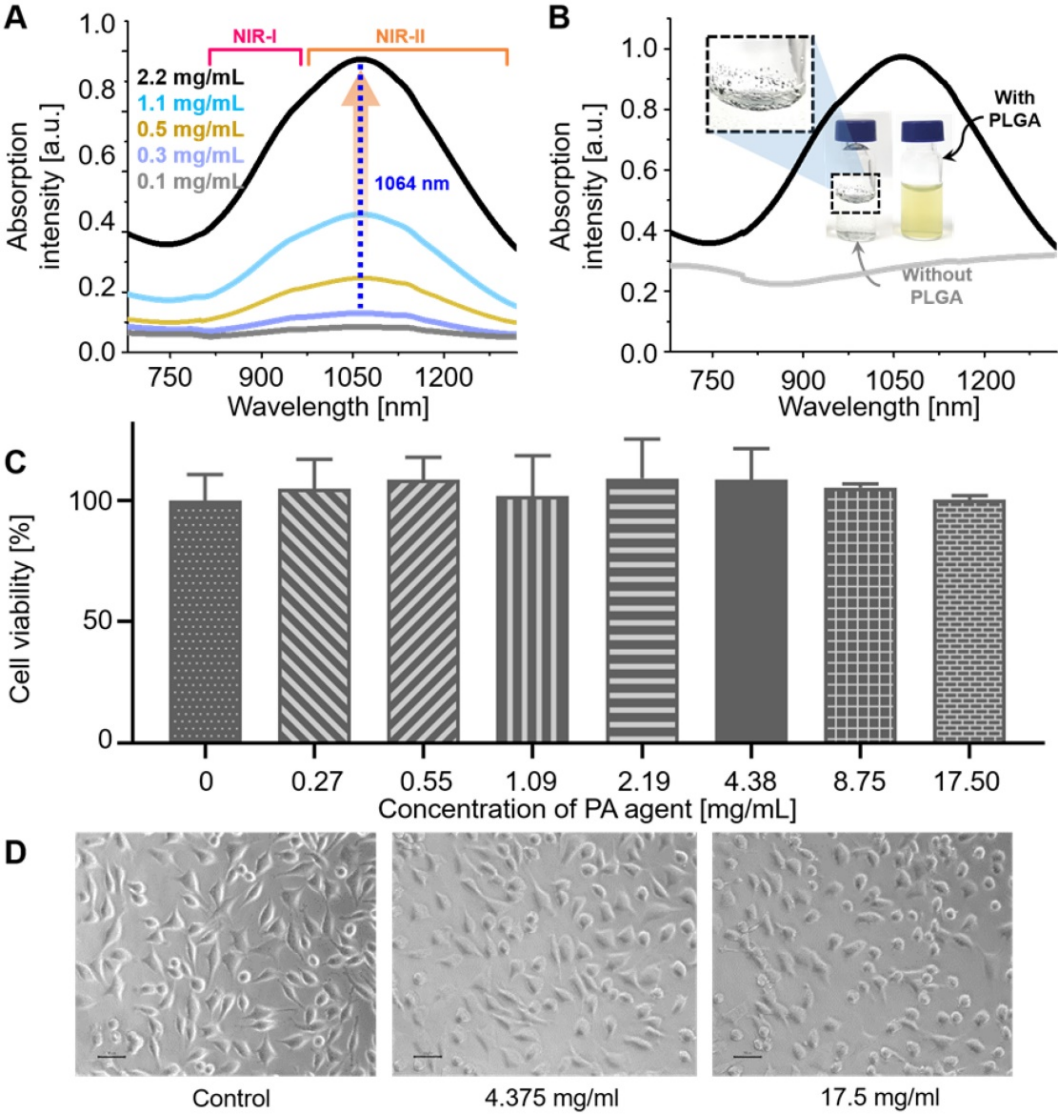
(A) NIR-I and -II absorption spectra of NiPNP measured at different concentrations from 0.1 to 2.2 mg/mL. The maximum absorption peak appears at 1064 nm (dotted blue). (B) NIR-I and -II absorption spectra of NiPNP (with PLGA, black) and the core Ni(II) complex (without PLGA, gray) in water. Without PLGA, the complexes were aggregated and precipitated in water and did not show the absorption spectrum when compared with NiPNP. Photographs of the metal complex (without PLGA, left) and the resulting NiPNP (with PLGA, right) in water. Cell viability of L929 mouse fibroblasts cells treated with NiPNP and phase contrast microscopic images. (C) Cells were treated with NiPNP at indicated concentration for 48 h and the amount of viable cells were measured by MTT kit. (D) Microscopic images after NiPNP incubated with cells (40 ×). Data are presented as the mean ± SD of three independent experiments. (Scale bar 30 µm) NiPNP, Ni(II) complex-containing polymeric nanoparticles; PLGA, poly(lactic-co-glycolic acid); TEM, transmission electron microscope;; NIR, near-infrared.

As designed, NiPNP showed NIR absorbance in the NIR-II region (Figure 2A) with the maximum absorption intensity at 1064 nm. The optical absorption of NIR-II at 1064 nm was about 257% higher than that of NIR-I at 700 nm. The absorption intensity at 1064 nm proportionally increased with the increase in the concentration of NiPNP from 0.1 to 2.2 mg/mL and the photographs of NiPNP revealed a yellowish green color that grew richer in proportion to the concentration (Figure S6). The contrast agent was considerably stable in water because of the PLGA shell that rendered dispersibility and biocompatibility in aqueous media. For example, we were still able to find the same absorption spectrum (Figure S7A) and particle size distribution (Figure S7B) after storing the contrast agent for 3 months at room temperature. The photograph taken after the storage was similar as before without severe aggregation (Figure S7C). Without PLGA, absorption in the NIR-II region was not completely developed (Figure 2B), and only aggregation of the complexes was observed (inset in Figure 2B). Only floating aggregates of the complex were observed on the surface of water (inset in Figure 2B) and the proper investigation of absorbance was thwarted, corroborating the crucial role of PLGA. The zeta potential of the contrast agent was found to be -30.22 mV, further supporting the colloidal stability of NiPNP in water (Figure S8).

### *In vitro* and *in vivo* toxicity

The cytotoxicity of the NiPNP was tested with L929 cell line *in vitro*. The NiPNP demonstrated low toxicity with over 90% cell viability from 0 to 17.5 mg/mL (Figure 2C). Furthermore, no significant cell damages were observed on microscopic images (Figure 2D) and live/dead florescent microscopic images (Figure S9). *In vivo* toxicity testing of NiPNP was also conducted. After intravenous injection of NiPNP, the white Balb/c mice behavior was observed for 1 month, but no symptoms of toxicity were observed. Three mice were sacrificed at 7 and 28 days, respectively, and the mice injected with PBS were prepared as the control group. After extracting the major organs (hearts, lungs, livers, kidneys, and spleens) of the mouse, the histological data were prepared according to the general hematoxylin and eosin (H & E) staining method (Figure S10). No specific organ damage or inflammation was found at 7 and 28 days, showing similar results as the control. In order to investigate the possibility of further toxicity, a complete blood count test and blood biochemistry were tested at 7 and 28 days after injecting NiPNP (100 mg / Kg) into white Balb/c mice. The measured values were shown in the normal range (Figure S11). Thus, toxicity test results indicate that the biocompatibility of NiPNP was high for at least 28 days and within the dose used. ICP-MS analysis method was also conducted to estimate remaining the NiPNP in the main internal organs 24 hrs after intravenous injecting the NiPNP (Figure S12). The remained Ni concentration in organs (kidney, lung, liver, spleen, and heart) were not significant compared with PBS injection data (NiPNP, 0.49 ± 0.02, 8.10 ± 5.13, 3.60 ± 3.11, 1.77 ± 1.03, and 0.53 ± 0.33 ng Ni per mg tissue; PBS 0.16 ± 0.13, 0.20 ± 0.30, 0.07 ± 0.05, 0.58 ± 0.72, and 0.42 ± 0.36 µg Ni per mg tissue).

### *In vitro* PA experiments and properties

**Figure 3:**
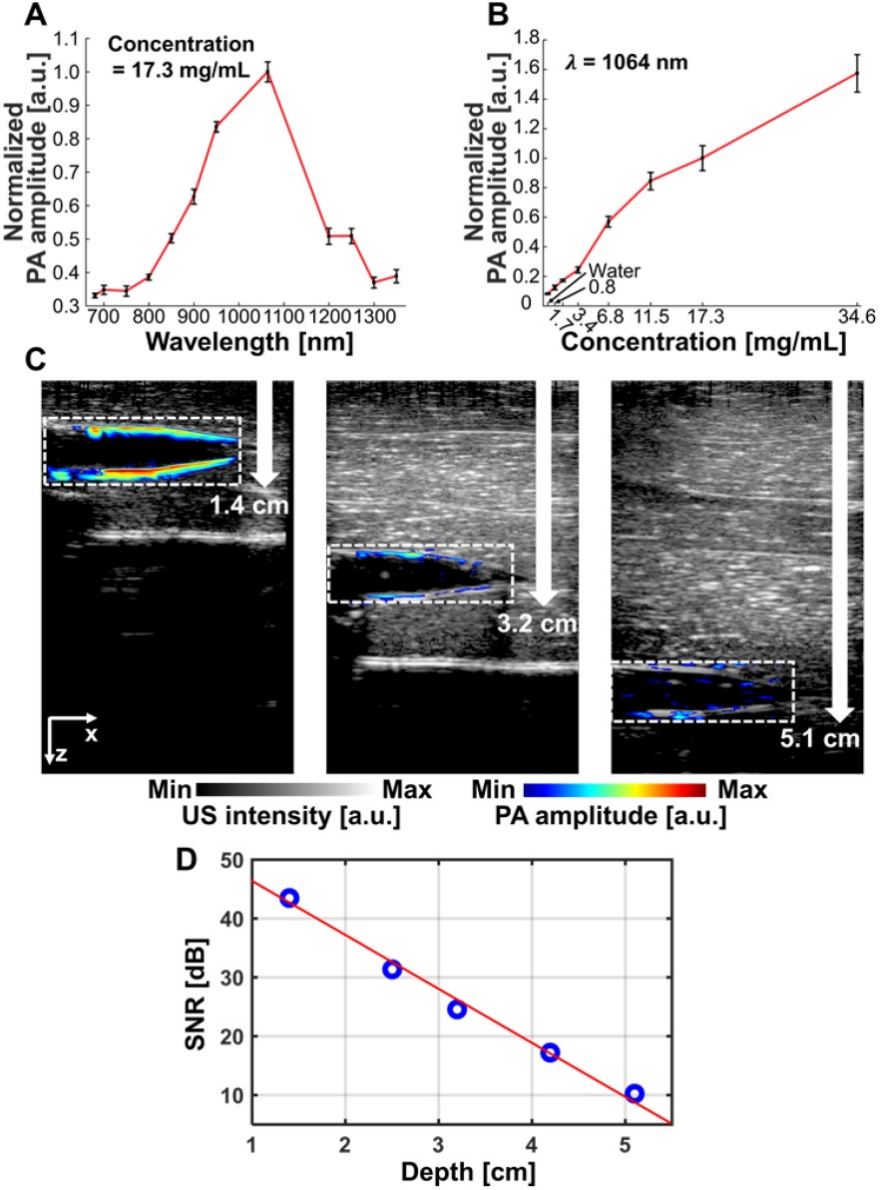
PA characteristics of the NiPNP *in vitro*. (A) PA spectrum of the NiPNP at 17.3 mg/mL concentration. (B) PA sensitivity of the NiPNP at various concentrations with a wavelength of 1064 nm. (C) PA/US overlaid images of the tube filled with NiPNP at different depths prepared with chicken breast tissues. (D) Quantification of PA SNR at different depths. PA, photoacoustic; US, ultrasound; NiPNP, Ni(II) complex-containing polymeric nanoparticles; SNR, signal-to-noise ratio.

The measured optical absorption intensity of NiPNP follows a Gaussian distribution with a peak at 1064 nm in the NIR-II region (Figure 2A). To observe the PA characteristics of NiPNP, we prepared 8 vessel mimicking tubes with different concentrations of the NiPNP and measured the PA signals using an AR-PAM system 10. As the NiPNP concentration increased, the PA amplitude also increased, and the strongest PA signals were generated by the 1064 nm wavelength in all different concentrations (Figure S13). For bioimaging in an animal model, the NiPNP concentration of 17.3 mg/mL was selected due to its high optical absorption and low toxicity with over 90% cell viability. The PA spectrum of the NiPNP with a concentration of 17.3 mg/mL was measured from 700 nm to 1350 nm, and confirmed that the PA spectrum (Figure 3A) is almost identical to the optical spectrum. The PA amplitude of NIR-II at 1064 nm was about 287% higher than that of NIR-I at 700 nm. In addition, the PA sensitivity of NiPNP at 1064 nm was measured at different concentrations (Figure 3B). PA signals have a linear relationship with concentration by default, but because they contain nonlinear components (e.g., response of the transducer and plastic tubes), it is difficult to represent a complete linear relationship in the experiment. However, looking at the minimum to maximum concentrations, overall linearity trend was identified. PA spectrum and PA sensitivity graphs were normalized to 1 at 1064 nm and 17.3 mg/mL, respectively. In addition, the PA response was obtained for 10 mJ/cm^2^ pulse energy at 1064 nm to verify the photostability of NiPNP with a concentration of 17.3 mg/mL (Figure S14). The light energy was appropriately selected based on the penetration depth of 1/e, assuming agents are placed in the tissue. The PA amplitude of the NiPNP shows a fairly stable result even while 3000 laser pulses (5 mins) were irradiated to the sample (Figure S14A). Also, NiPNP before laser irradiation and NiPNP after laser irradiation of 3000 shots were confirmed by TEM, showing little change in particle shape (Figure S14B). After 3000 shots, the PA signal was reduced. To explore the feasibility of using NiPNP as a deep tissue PAI contrast agent, the overlaid PA/US images with NiPNP were acquired at various depths in chicken breast tissues (Figures 3C, D). In the overlaid PA/US images, the PA signal in pseudo color represents the absorption of light in the tube filled with the NiPNP, and the US signal in a gray scale represents the morphological structure of the tube and surrounding chicken tissues (Figure 3C). The reason why the PA signal is observed only at the boundary of the tube is related to the limited bandwidth of the transducer 63. PA signals generated inside the tube are mainly low frequency components, and PA signals generated at the boundary are mainly high frequency components. However, because the bandwidth of the transducer used in the experiments was relatively high, most low-frequency signals generated inside the tubes could not be detected, which is a typical phenomenon of the bandwidth limited PA detection. The maximum detectable penetration depth for the PA signal was ~5.1 cm with the SNR of 10.2 dB. As the imaging depth increases, the SNR of the PA signal decreases exponentially, and the measured 1/e decay was 0.95 cm (Figure 3D). The experimentally measured PAI depth is 5.3 times greater than the optical penetration depth.

### *In vivo* and *ex vivo* PA experiments

**Figure 4:**
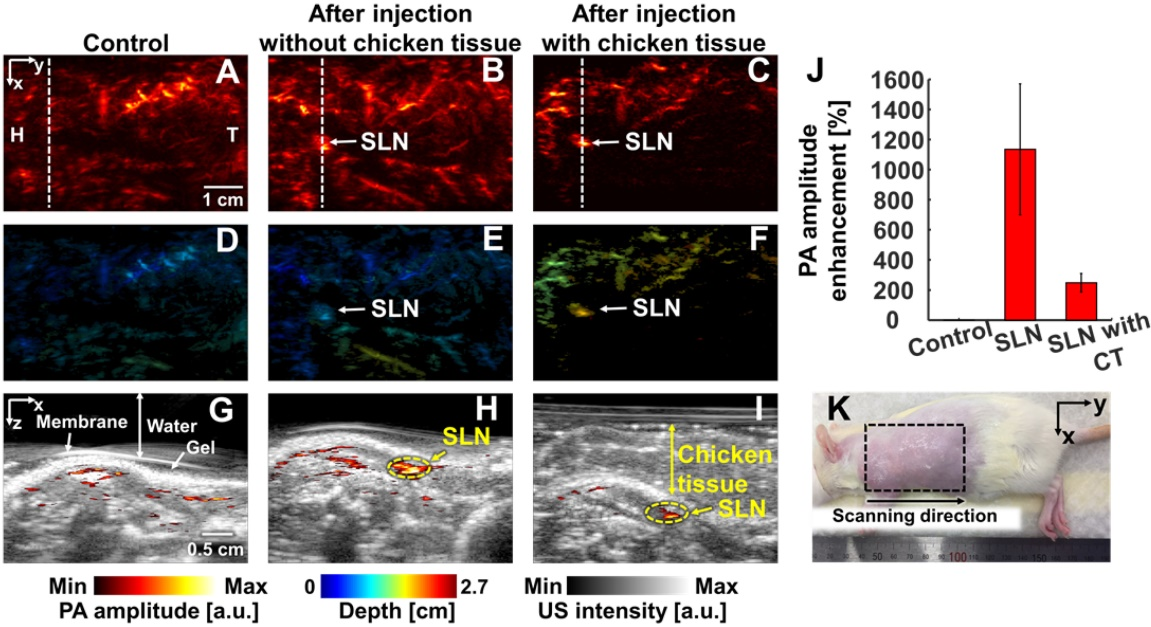
*In vivo* PA imaging of rats SLN (n = 3) using a 1064 nm laser excitation. (A) Before, (B) after without chicken tissue and (C) after with chicken tissue injection of NiPNP PA MAP images. (D-F) obtained by applying depth encoding processing to panel (A-C), respectively. (G-I) Two-dimensional PA/US overlaid images obtained along the white dashed lines in panel (A-C), respectively. (J) PA enhancement comparison before injection of NiPNP, after injection of NiPNP without chicken tissue and after injection of NiPNP with stacking the chicken tissue. (K) Photograph of the rat before *in vivo* PA imaging of SLN. Error bar denotes the standard error of three experiments. PA, photoacoustic; US, ultrasound; NiPNP, Ni(II) complex-containing polymeric nanoparticles; MAP, maximum amplitude projection; SLN, sentinel lymph node; H, head; T, tail; CT, chicken tissue.

**Figure 5:**
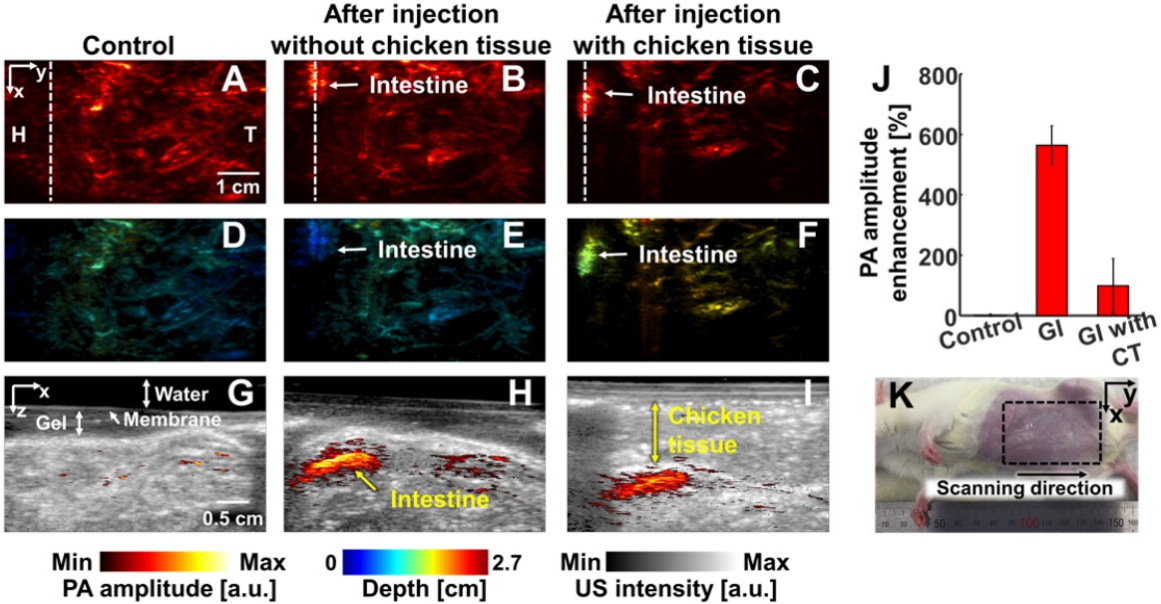
*In vivo* PA imaging of rats GI tract (n = 3) using a 1064 nm laser excitation. (A) Before, (B) after without chicken tissue and (C) after with chicken tissue injection of NiPNP PA MAP images. (D-F) obtained by applying depth encoding processing to panel (A-C), respectively. (G-I) Two-dimensional PA/US overlaid images obtained along the white dashed lines in panel (A-C), respectively. (J) PA enhancement comparison before injection of NiPNP, after injection of NiPNP without chicken tissue and after injection of NiPNP with stacking the chicken tissue. (K) Photograph of the rat before *in vivo* PA imaging of GI tract. Error bar denotes the standard error of three experiments. PA, photoacoustic; US, ultrasound; NiPNP, Ni(II) complex-containing polymeric nanoparticles; MAP, maximum amplitude projection; GI, gastrointestinal; H, head; T, tail; CT, chicken tissue.

**Figure 6:**
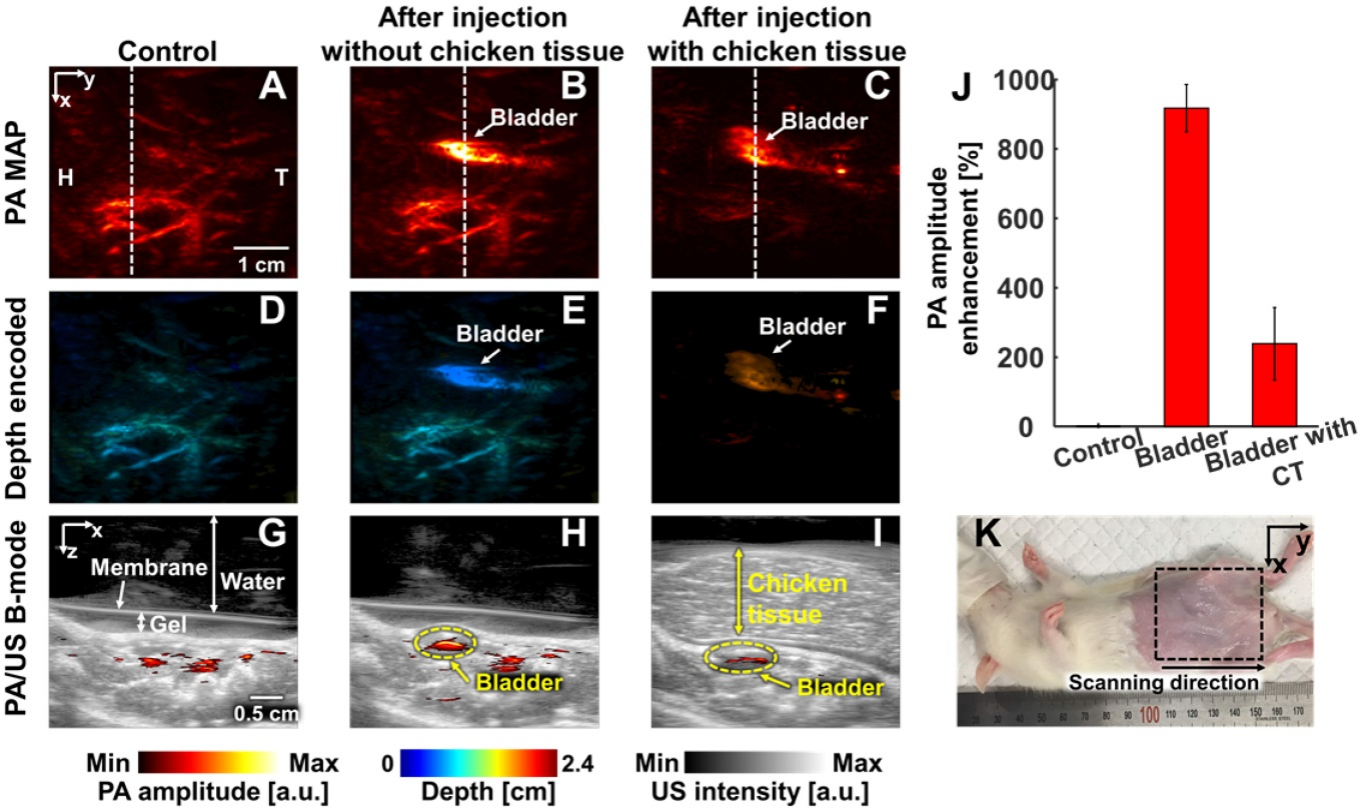
*In vivo* PA imaging of rats bladder (n = 3) using a 1064 nm laser excitation. (A) Before, (B) after without chicken tissue and (C) after with chicken tissue injection of NiPNP PA MAP images. (D-F) obtained by applying depth encoding processing to panel (A-C), respectively. (G-I) Two-dimensional PA/US overlaid images obtained along the white dashed lines in panel (A-C), respectively. (J) PA enhancement comparison before injection of NiPNP, after injection of NiPNP without chicken tissue and after injection of NiPNP with stacking the chicken tissue. (K) Photograph of the rat before *in vivo* PA imaging of bladder. Error bar denotes the standard error of three experiments. PA, photoacoustic; US, ultrasound; NiPNP, Ni(II) complex-containing polymeric nanoparticles; MAP, maximum amplitude projection; H, head; T, tail; CT, chicken tissue.

We conducted *in vivo* experiments on rats to confirm that NiPNP with strong absorbance at 1064 nm could be used as the PA agents in deep tissues (Figures 4-6). We imaged clinically important SLN (n = 3), GI tract (n = 3) and cystography (n = 3) 42. We adopted the rat model because the skin of a rat is more structurally similar to human tissue than other rodents. Figure 4 shows the PA/US images of SLNs, Figure 5 shows those of GI tracts, and Figure 6 shows those of bladders. Panels A-C in Figure 4-6 represents the PA maximum amplitude projection (MAP) images acquired pre-NiPNP injection, post-NiPNP injection without chicken tissues, and post-NiPNP injection with chicken tissues, respectively. Panels D-F of Figure 4-6 are obtained by applying a depth encoded image processing method to the panels A-C, respectively. Panels G-I in Figure 4-6 represent the 2D depth-resolved PA/US images obtained along the white dashed lines in the panels A-C, respectively. Panel J in Figure 4-6 shows the quantification results of PA amplitude enhancement at the NiPNP injection sites (pre-injection, post-injection without chicken tissues, and post-injection with chicken tissues). The imaging region is indicated by the black dashed boxes in the animal photographs as shown in Figure 4K-6K. The PA amplitude enhancement was calculated as the percentage increase in the PA amplitudes pre- and post-NiPNP injection (i.e., (PA_after_ -PA_before_)/ PA_before_ × 100) at the SLNs, GI tracts, and bladders. The error bar denotes the standard error for n = 3.

PAI of sentinel lymph nodes: We photoacoustically imaged SLNs in the rat model before injecting the NiPNP. It was not visible in the PA image due to the optical transparency in the control image. At 24-hrs-post-injection, the SLN was clearly visualized in the PA image and showed a PA amplitude enhancement of 1135 ± 434% compared to control SLN region. In addition, even after a 1.2-cm-thick chicken breast tissue was placed on the rat, the SLN was visible in the PA images with a PA amplitude enhancement of 248 ± 62%. The depth-encoded PA images and the overlaid PA/US B-mode demonstrated the clear visibility of SLN even at a depth of ~1.5 cm with the chicken breast tissues. To verify the *in vivo* PA SLN imaging results, the draining lymph nodes and control lymph nodes were excised from the axillary region of rats and were imaged using the clinical PA/US imaging system. In *ex vivo* PAI, the only draining SLN is photoacoustically visualized with great PA amplitude enhancement (2697 ± 827%, Figure S15). To confirm the efficiency of NIR-II PAI of NiPNP *in vivo*, NIR-I PAI results were examined (Figure S16). After injection of NiPNP, a weak SLN signal was observed in PA image obtained by irradiation with 800 nm laser source. Compared with the control PA image, PA amplitude enhancement was 115% and 18% before and after stacking chicken tissues*.* We confirm that the SNR in the NIR-II is about 6.7 dB higher than that in the NIR-I *in vivo*. Despite the signal observed in the SLN, the NIR-I laser is absorbed more by blood vessels and other tissues than the NIR-II laser, making it difficult to see the clear contrast of the SLN compared to the surroundings.

PAI of GI tracts: We noninvasively and photoacoustically imaged the GI tracts of the small animals with oral administration of NiPNPs. The PA images of the GI tract were acquired before and after 2 hrs post-NiPNP oral administration. Because the rat was starved for 2 days before acquiring the PA image, the PA signals were scarcely visible in the GI tract of the control PA image. In contrast, the GI tract was clearly visible in the PA images obtained post-NiPNP injection with the enhanced PA amplitude (564 ± 63% and 99 ± 90%, respectively) and with/without stacking of the chicken tissues on the abdomen of the rat. In the depth-encoded images and the PA/US overlaid B-mode images, we confirmed that the PA signals enhanced by the NiPNP were visible at a depth of 1.5 cm, including the chicken tissues.

PAI of bladders: In the third application, we imaged the bladders of the small animals with a transurethral injection of NiPNPs noninvasively and photoacoustically. In the PA image acquired before injecting the NiPNP, the bladder is invisible due to transparency at 1064 nm. However, the bladder is clearly visible in the PA images acquired post-NiPNP injection. The calculated PA amplitude enhancements without and with the chicken tissues placed on the rat's abdomen are 918 ± 68% and 238 ± 104%, respectively. The overlaid PA/US B-mode and depth-encoded PA images enhanced by the NiPNP injected into the bladder show that the PA signals are generated at depths of about 1.7 cm, including the chicken breast tissues. Additionally, bladder PA image of a rat was acquired with higher laser power (~ 66 mJ/cm^2^) to demonstrate the potential of NiPNP as a PA contrast agent in deeper tissues (Figure S17). As a result, a PA image was obtained from the bladder at a depth of 3.4 cm including chicken tissues, confirming that it was about 200% PA amplitude enhancement compared to the control.

## Discussion

The key of deep tissue PAI is to develop agents that are safe and have strong and specific optical absorption properties in the NIR-II. The newly developed contrast agent, namely NiPNP, meets all the above two criteria and we confirmed the feasibility of the NiPNP as a PA contrast agent *in vitro, in vivo, and ex vivo*. The polymeric particles were facilely prepared via nanoprecipitation using the nickel(II) bis(dithiolene) complex and PLGA. In brief, when precipitated under aqueous conditions in the presence of PLGA, the BDN particles were encapsulated by the surfactant polymer through physical interaction such as hydrophobic force and stabilized without further coagulation, leading to the formation of core-shell type NiPNPs. As designed, the core-shell particle not only showed biocompatibility and structural stability but also had strong optical absorption in the NIR-II window. The optical absorption at 1064 nm was about 257% higher than that at 700 nm. In particular, cell viability with over 90% at 17.5 mg/mL and *in vivo* cytotoxic test including histological data and blood analysis show low toxicity of NiPNP and applicability to future clinical applications. Based on these results, the dose of *in vivo* PA experiment was determined to be 17.3 mg/mL and offered two advantages: excellent biocompatibility and PA contrast.

The PA amplitude characteristics of NiPNP followed the Gaussian distribution in the NIR-II window with a peak at 1064 nm. The PA amplitude at 1064 nm was about 287% higher than that at 700 nm. The maximum depth of the detectable PA signal at 1064 nm in biological tissues was about 5.1 cm. Unfortunately, the laser power used in the *in vitro* PA experiment was limited to 40 mJ/cm^2^, corresponding to 40% of the ANSI limit, due to the limitation of the experimental environment. We expected that increasing the laser power to the ANSI safety limit will increase the imaging depth. Although the laser power was not sufficient, our results shows that the NiPNP can serve as a PA contrast agent for clinical application requiring large image depths.

The potential of using NiPNP for preclinical and clinical investigation was demonstrated via *in vivo* and *ex vivo* PAI of SLNs, GI tracts, and bladders in rats, thus could be translated for human clinical evaluation 64-70. Note that the NiPNP concentration for *in vivo* experiments was performed at 17.3 mg/mL based on cell viability results. To further enhance the possibility of clinical translation, we used the clinical PA/US imaging system with a 1064-nm laser 71, 72. The three applications each require PAI with extrinsic agents for the following clinical significance: (1) The SLN biopsy, a prognostic indicator of cancer metastasis, using radioactive colloids and colored dyes is a representative method for the staging of metastatic cancer 16. Ionizing radiation from SLN biopsies is known to have no side effects to patients and physicians if properly followed the regulations, but may be adversely affected by radiation exposure when the does amount, the exposure time, the distance from the radiation source and the shielding are misused 73 . As an alternative to avoid this potential risk, the PAI using extrinsic agents has proven to be a suitable tool for SLN visualization 4, 42, 68. (2) The GI imaging plays an important role in the diagnosis and treatment of GI related diseases. In particular, intestinal motility disorders cause variety of bowel disorders such as bacterial growth, irritable bowel syndrome, and in many diseases even severe side effects, such as thyroid disorders and diabetes 13, 74. The X-ray and computed tomography are the leading imaging tools for GI observation. However, these imaging devices use ionizing radiation. Safe, non-invasive and non-ionized PA imaging can be a great alternative imaging tools for GI observation. (3) Bladder imaging is essential for monitoring diseases such as vesicoureteral reflux, cystitis, glomerulation, and bladder cancer. In particular, cystography is the gold standard imaging method for diagnosing bladder related diseases using radio-opaque contrast agents. However, this technique does not have high sensitivity and can negatively affect the prognosis of a patient due to the ionizing radiation used 75. Comparing the PA images obtained before and after NiPNP injection in *in vivo* applications, we confirmed that the PA signals obtained at the SLNs, GI tracts, and bladders were improved by up to 1135 ± 434%, 564 ± 63% and 918 ± 68%, respectively. In addition, *in vivo* PA images of the rats obtained by increasing the tissue depth using chicken breasts in SLNs, GI tracts and bladders showed 248 ± 62%, 99 ± 90% and 238 ± 104% enhanced PA signal, respectively.

Studies to enhance the PA signals using NIR-II absorbers have recently been published in other studies. However, as mentioned in Introduction section, in most studies, the feasibility of deep tissue PAI was not confirmed, or the light absorption properties of the agents slightly deviated from 1064 nm, which limits the efficiency of the laser. Table S1 shows brief comparison of the major NIR-II PA agents. This study is not only limited to the development of agents with the highest optical absorption properties at 1064 nm, but also it is significant that we have experimentally validated deep tissue PAI using rats with a similar tissue structure to human. Additionally, although NiPNP was verified to be nontoxic, ICP-MS results 24 hours after NiPNP injection confirmed that NiPNP accumulated in major organs. However, ICP-MS results also confirmed that NiPNP could be released as nickel was detected in the kidney. Further research on long-term release due to biodegradation of the particles will be made in subsequent studies.

## Conclusion

In this study, we proposed a nickel-based PA agent with sharp peak optical absorption at 1064 nm and verified its potential as a PA contrast agent through various experiments. We have also demonstrated the potential of NiPNP to be used in future clinical applications as a PA contrast agent by successfully obtaining *in vivo* deep tissue PAI using the clinically viable imaging system. These results suggest that the NiPNP would play an effective role as a PA contrast agent, which are required for the diagnosis of various diseases related to deep biological organs.

## Supplementary Material

Supplementary figures and table.

## Abbreviations

PAI: photoacoustic imaging
PA: photoacoustic
US: ultrasound
SNR: signal-to-noise ratio
GI: gastrointestinal
NIR: near-infrared
NiPNP: nickel dithiolene-based polymeric nanoparticle
PLGA: poly(lactide-*co*-glycolide)
FT-IR: Fourier-transform infrared
DI: deionized
TEM: transmission electron microscopy
AR-PAM: acoustic-resolution photoacoustic macroscopy
ANSI: american national standard insititute
SLN: sentinel lymph node
MAP: maximum amplitude projection
