# Supplementary material for: Deep tissue photoacoustic imaging of nickel(II) dithiolene-containing polymeric nanoparticles in the second near-infrared window
Source: Theranostics. 2020 Jan 22;10(6):2509–21. doi: 10.7150/thno.39403 (PMC7052900; doi:10.7150/thno.39403)
Supplement: Supplementary file 1 — Supplementary figures and table. [file thnov10p2509s1.pdf]

Supplementary Files

Deep tissue photoacoustic imaging of nickel(II) dithiolene-containing polymeric nanoparticles in the second near-infrared window

Byullee Park[a,†]*, Kyung Min Lee*[b,e,†]*, Suhyeon Park*[c]*, Misun Yun*[d]*, Hak-Jong Choi*[d]*, Jeesu Kim*[a]*,*

Changho Lee[c,e,]**, Hyungwoo Kim*[f,]**, and Chulhong Kim*[a,]**

†These authors contributed equally to this work

[a]Departments of Creative IT Engineering, Electrical Engineering, and Mechanical Engineering, Pohang University of Science and Technology (POSTECH), 77 Cheongam-ro, Pohang 37673, Republic of Korea. E-mail: chulhong@postech.edu

[b]Department of Materials Science and Engineering, College of Engineering, Seoul National University, Seoul 08826, Republic of Korea.

[c]Interdisciplinary Program of Molecular Medicine, Chonnam National University, 77 Yongbong-ro, Buk-gu, Gwangju 61186, Republic of Korea.

[d]Microbiology and Functionality Research Group, World Institute of Kimchi, 86 Kimchi-ro, Gwangju 61755, Republic of Korea.

[e]Department of Nuclear Medicine, Chonnam National University Medical School & Hwasun Hospital, 264, Seoyang-ro, Hwasun-eup, Hwasun-gun, Jeollanam-do 58128, Republic of Korea. E-mail: ch31037@jnu.ac.kr

[f]School of Polymer Science and Engineering, Chonnam National University, 77 Yongbong-ro, Buk-gu, Gwangju 61186, Republic of Korea. E-mail: kimhw@jnu.ac.kr

KEYWORDS: Photoacoustic imaging, Deep tissue imaging, Nickel dithiolene complex, Polymeric nanoparticle, Second near-infrared window

Figure S1. Schematics of an acoustic-resolution photoacoustic microscopy for (a) a reflection mode and (b) a transmission mode.

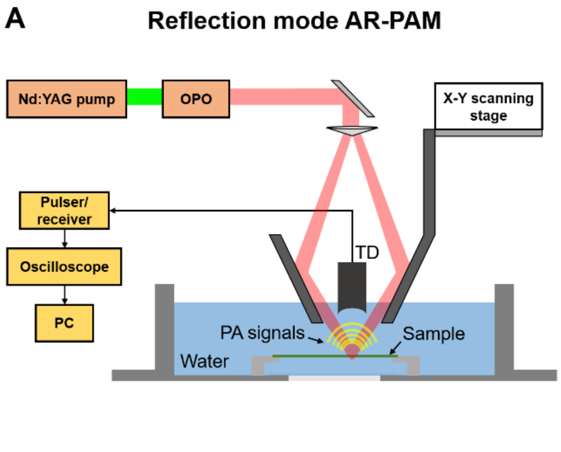

Figure S2. Schematic of clinical PA/US imaging system.US, ultrasound; TR, transducer; PC, personal computer; FB, fiber.

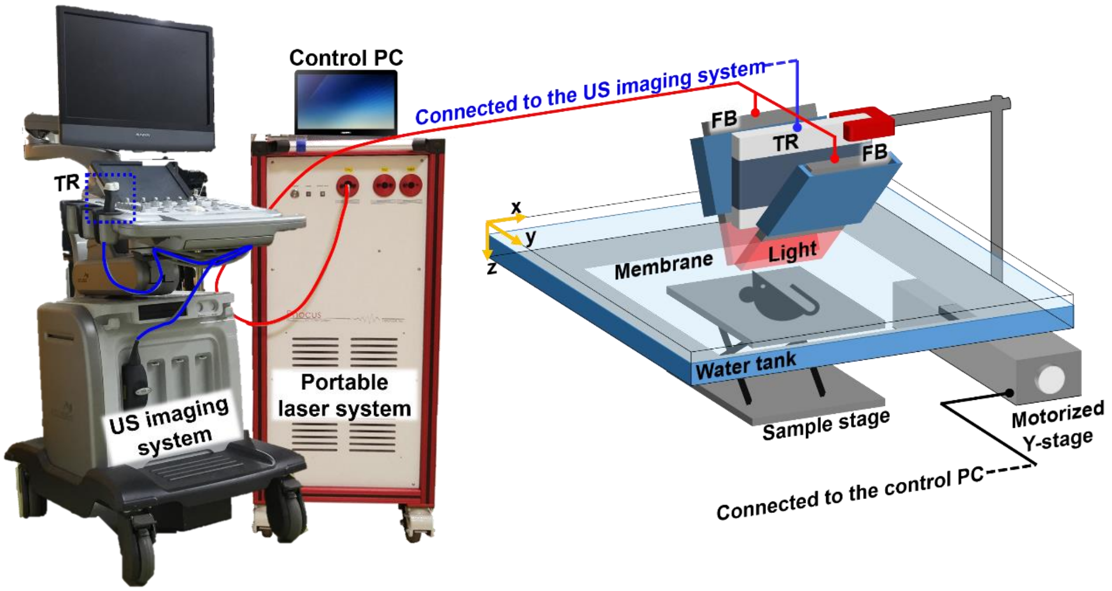

Figure S3. In vitro PA imaging of NiPNP in biological tissues. Photographs of (A) a tube filled with NiPNP and (B) a stack of chicken tissue on top of it. PA, photoacoustic; NiPNP, Ni(II) complex-containing polymeric nanoparticles.

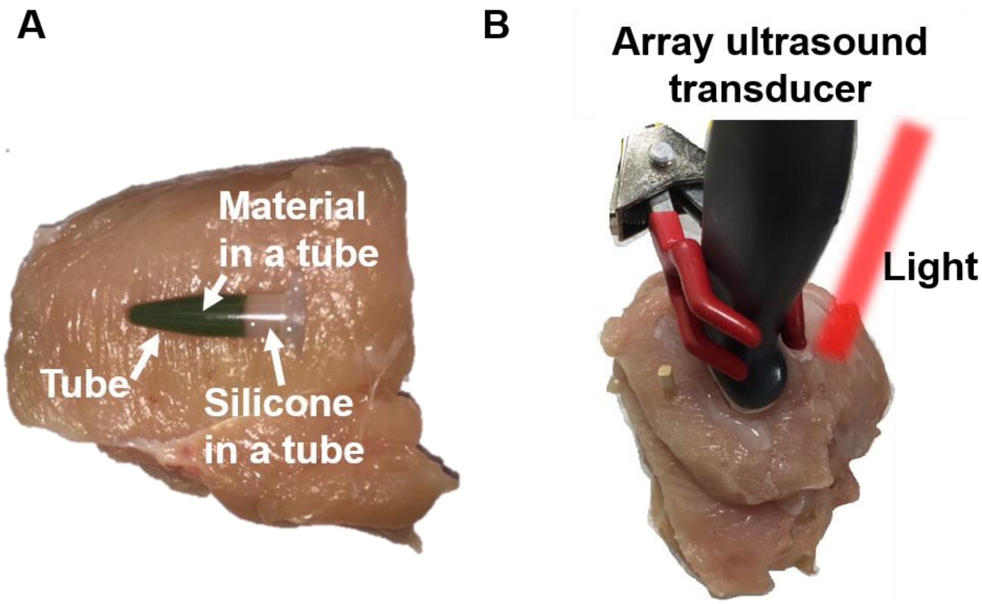

Figure S4. The EDS data of NiPNP. The inset table shows the weight and atomic composition of elemental C, S, O, and Ni in NiPNP.

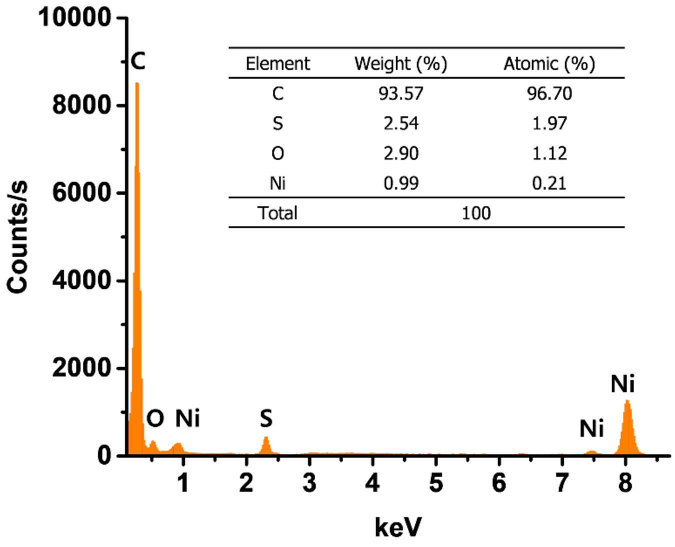

Element	Weight (%)	Atomic (%)
C	93.57	96.70
S	2.54	1.97
O	2.90	1.12
Ni	0.99	0.21
Total	100	

Figure S5. FT-IR spectra of PLGA (sky blue), BDN (olive), and NiPNP (black). The olive asterisks in the black spectrum at 1597 and 1351 cm^{-1} reveals the inclusion of BDN in PLGA in NiPNP.

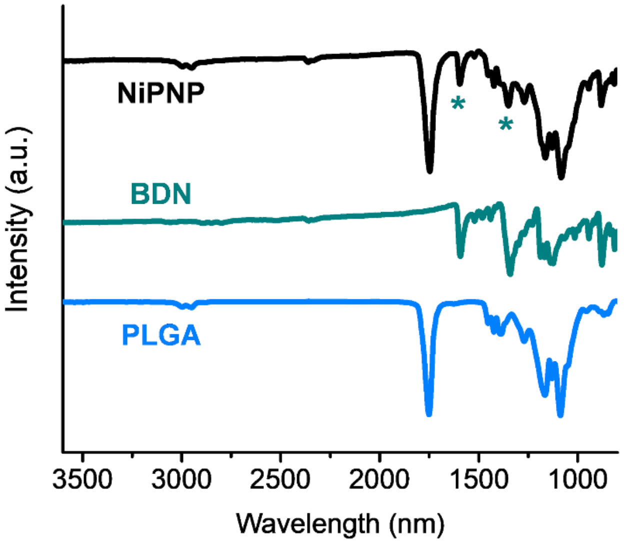

Figure S6. (A) The absorption maximum peak of NiPNP which was observed at 1064 nm linearly increased in proportion to the concentration of material. (B) Photographs of solutions of NiPNP in DI water at different concentrations from 0.1 to 2.2 mg/mL.

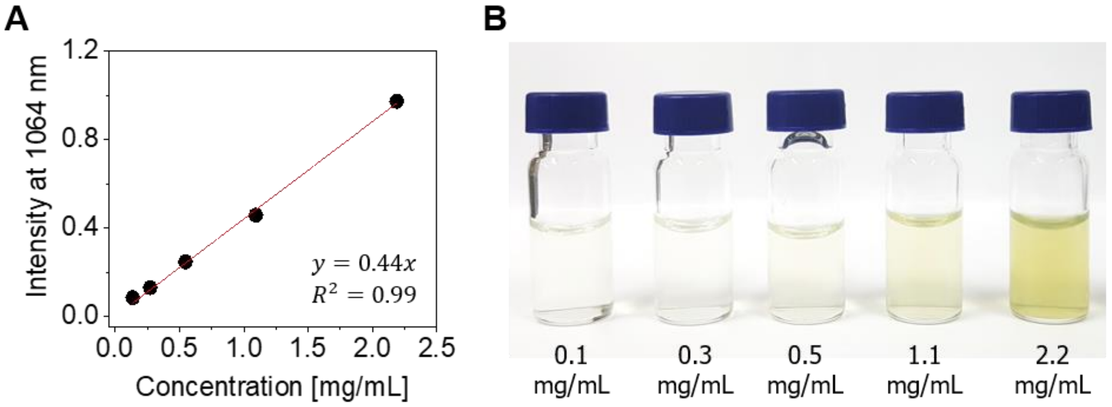

Figure S7. Stability of NiPNP in water (2.2 mg/mL). The absorption spectrum (A) and particle size distribution (B) of the sample did not change for 3 months at room temperature. (C) Photograph of NiPNP in water (2.2 mg/mL) after the 3-month storage at room temperature.

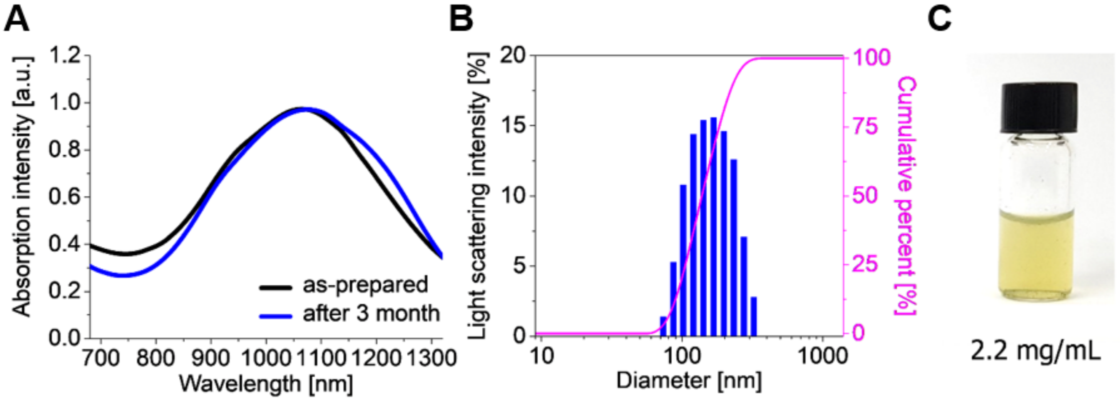

Figure S8. Zeta potential of NiPNP measured by a zeta potential meter.

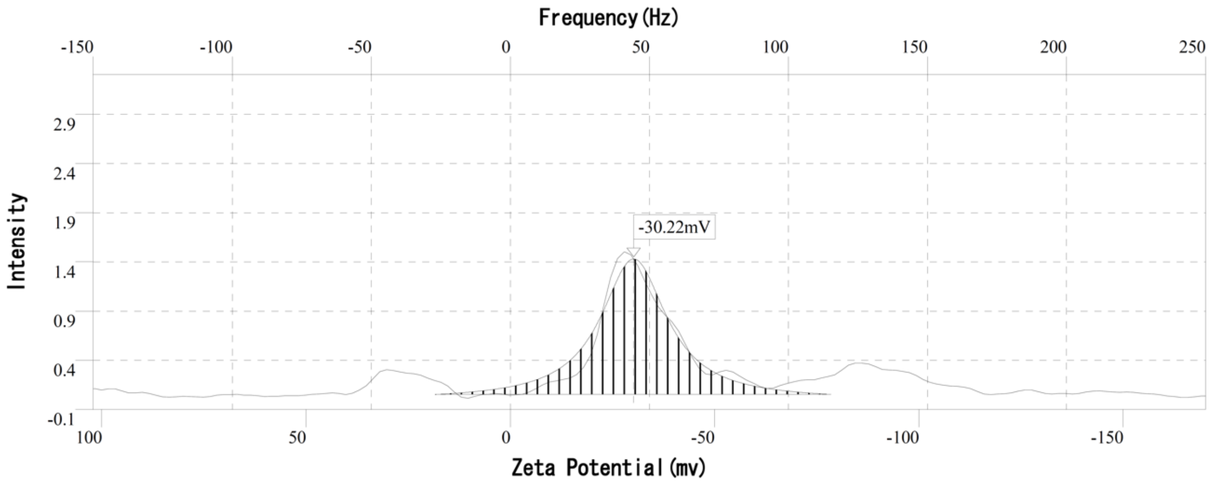

Figure S9. *In vitro* live/dead assay with NiPNP. (Scale bar = 100 μm)

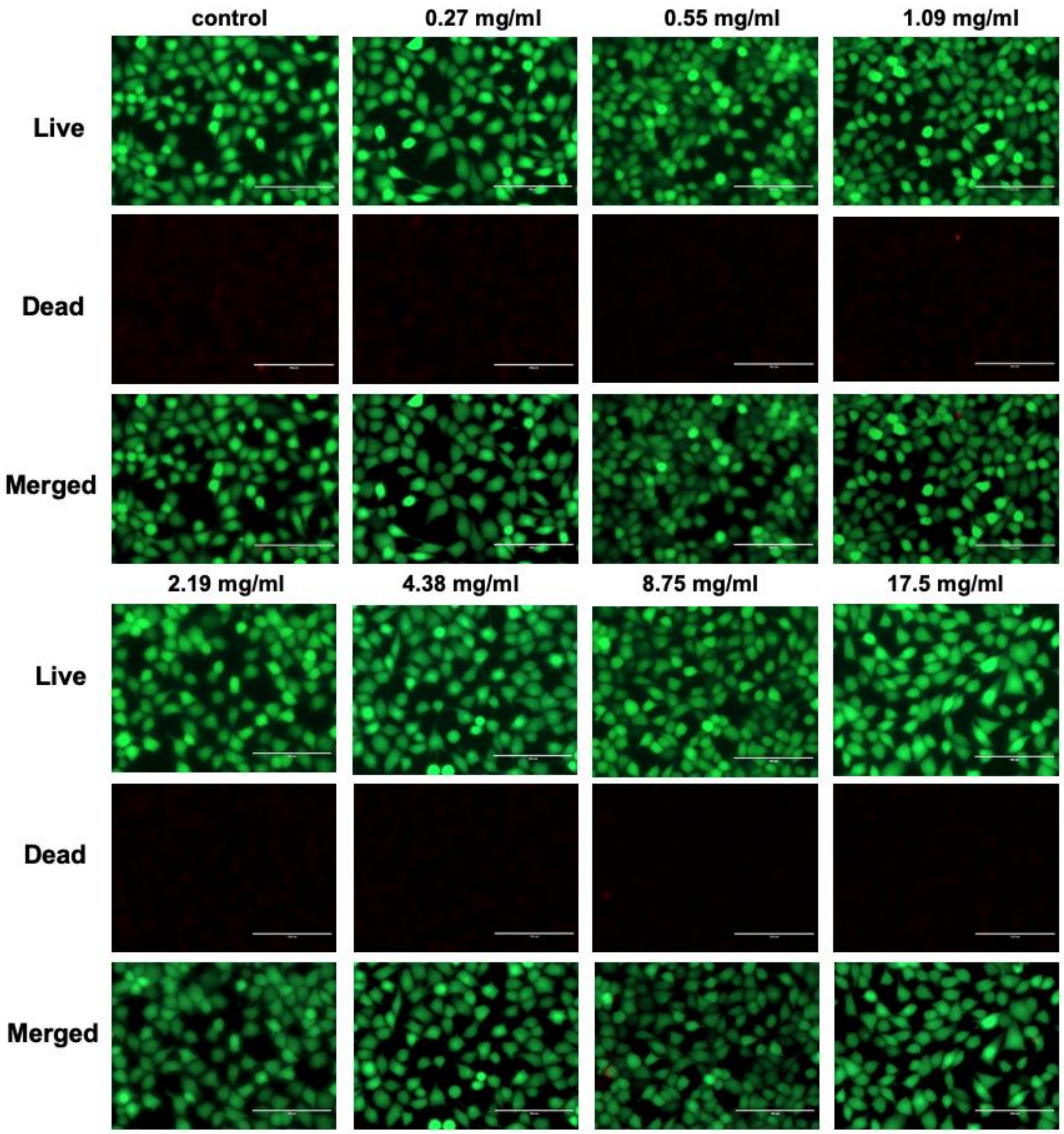

Figure S10. *In vivo* toxicity test of NiPNP with histological analysis. (Scale bar = 100 μm)

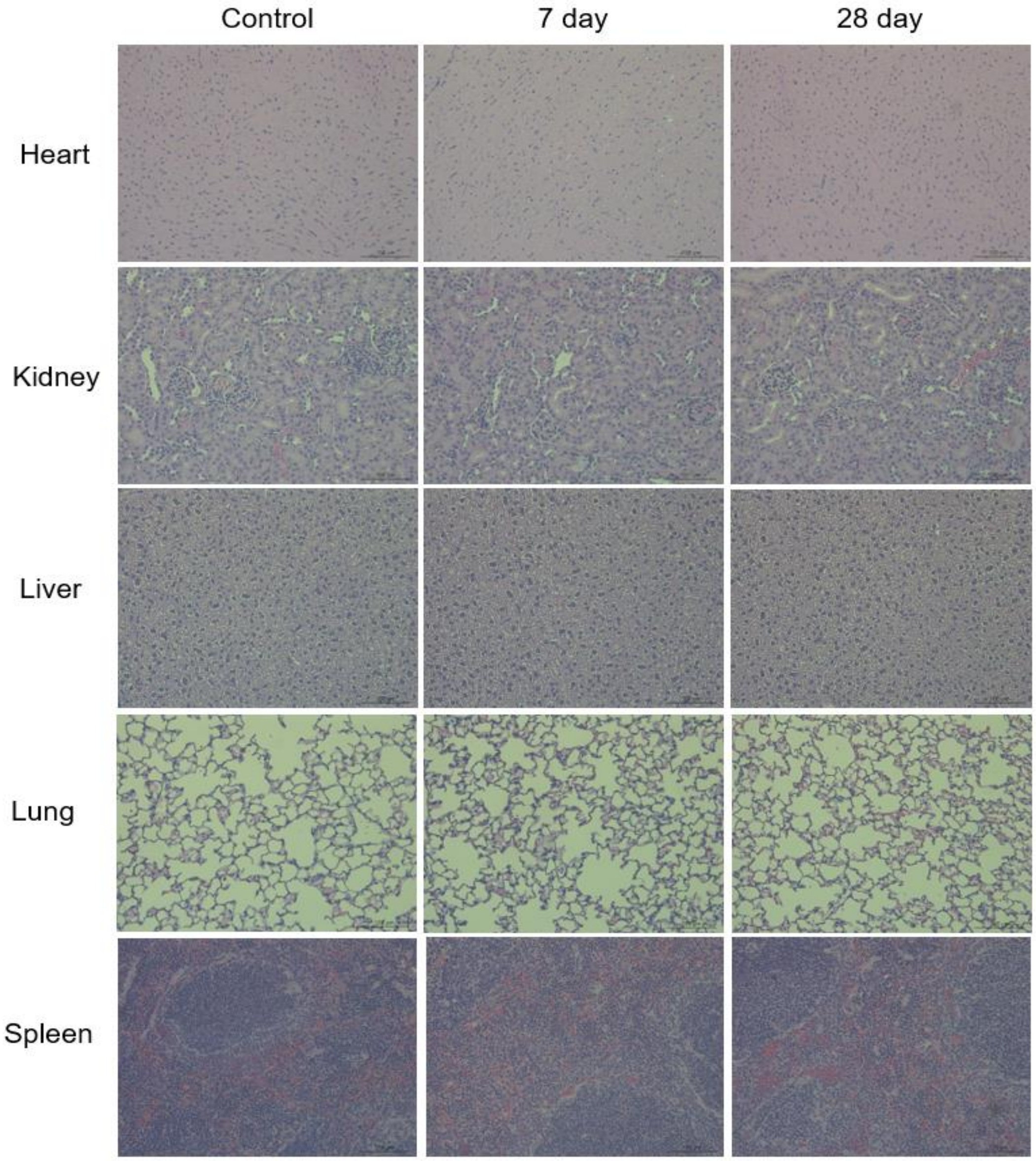

Figure S11. *In vivo* toxicity study of NiPNP with blood biochemistry assay.

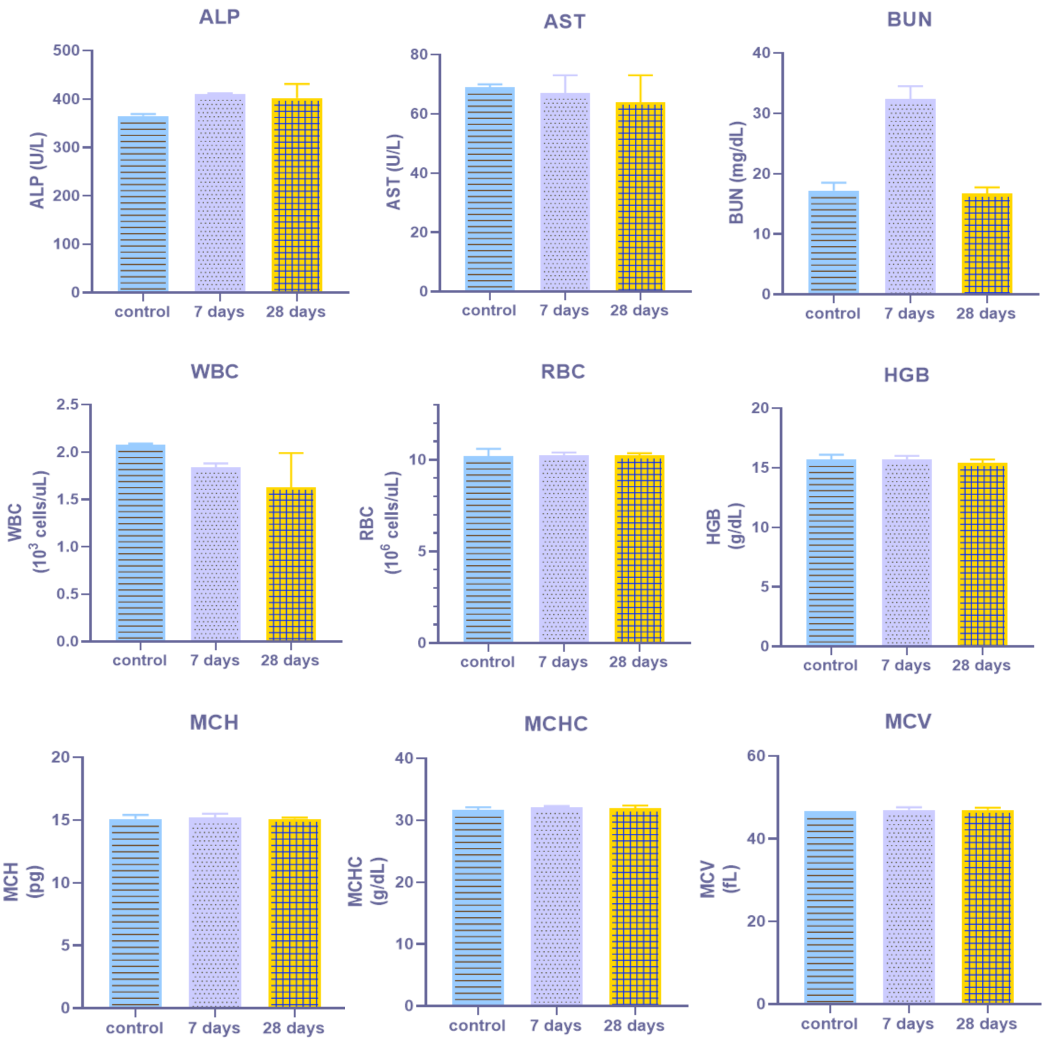

Figure S12. ICP-MS result of Ni in mice organs after 24 h of NiPNP and PBS injection.

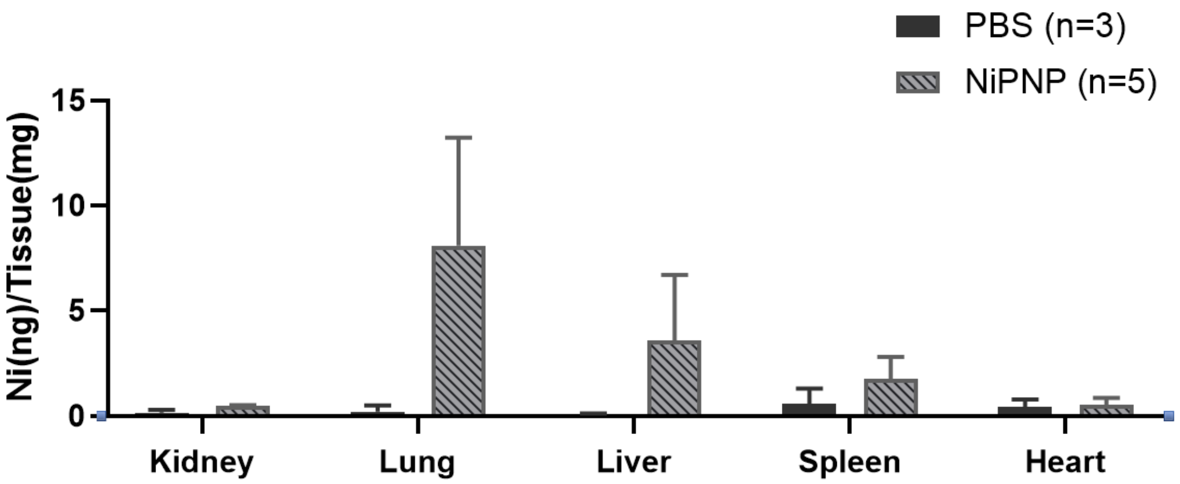

Figure S13. (A) Before normalization and (B) after normalization of PA spectra of the NiPNP measured at different concentrations from 0 to 34.66 mg/mL. The maximum absorption peak appears at 1064 nm. PA, photoacoustic; NiPNP, Ni(II) complex-containing polymeric nanoparticles.

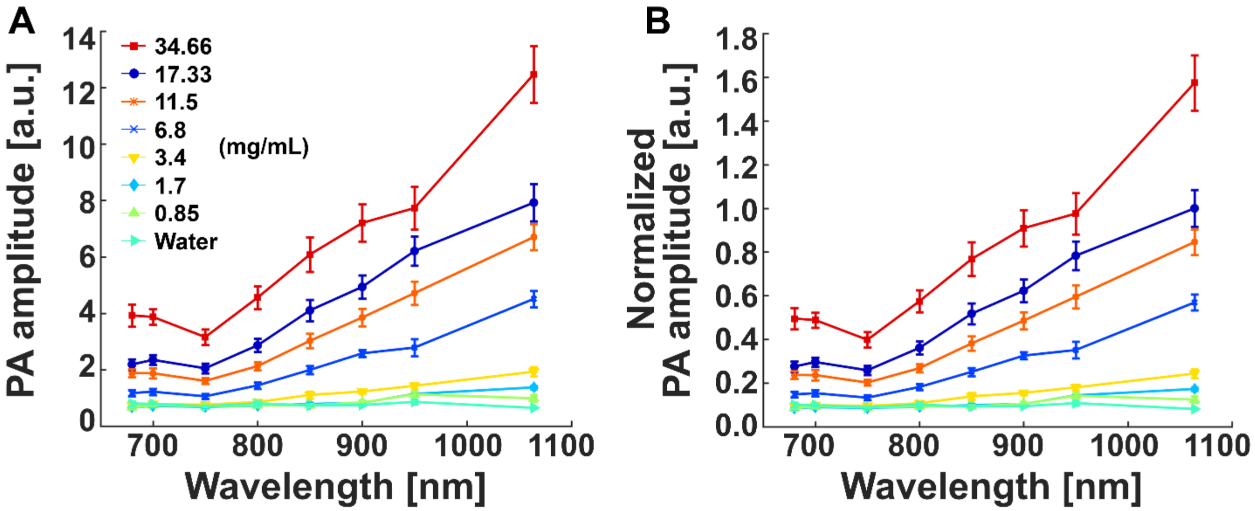

Figure S14. Photoacoustic characteristics of NiPNP. (A) The stability of the PA responses of NiPNP depending on the number of irradiated laser pulses onto the NiPNP. TEM images of NiPNP (B) before laser irradiation and (C) after laser irradiation of 3000 shots. PA, photoacoustic; NiPNP, Ni(II) complex-containing polymeric nanoparticles; TEM, transmission electron microscopy. Scale bar = 100 nm.

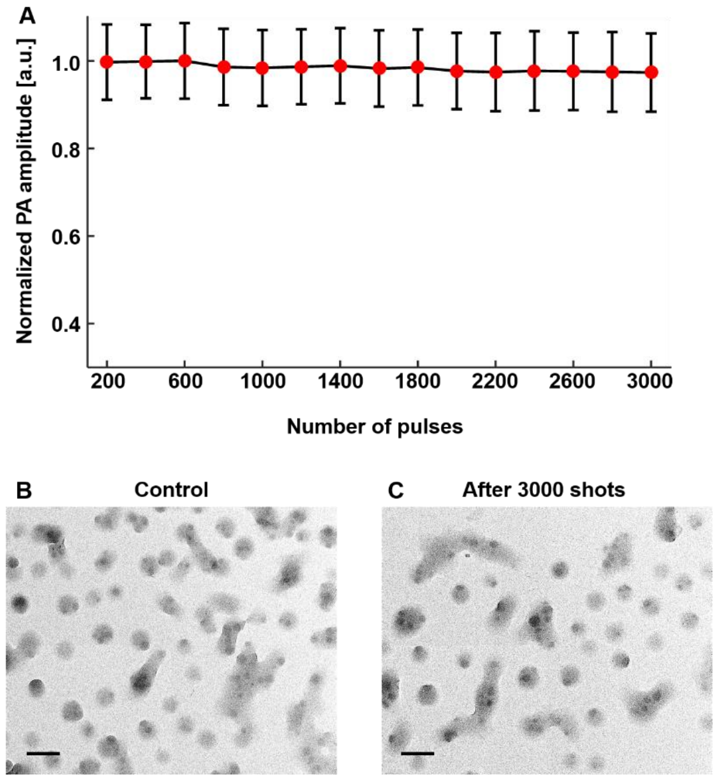

Figure S15. Photograph (A) and PA MAP image (B) of the excised LNs after injection NiPNP (red dashed boxes) and the normal LNs (blue dashed boxes) (n = 3). (C) PA amplitude enhancement of excised SLNs. Error bar denotes the standard error of three experiments. PA, photoacoustic; MAP, maximum amplitude projection; NiPNP, Ni(II) complex-containing polymeric nanoparticles; LN, lymph node; SLN, sentinel lymph node.

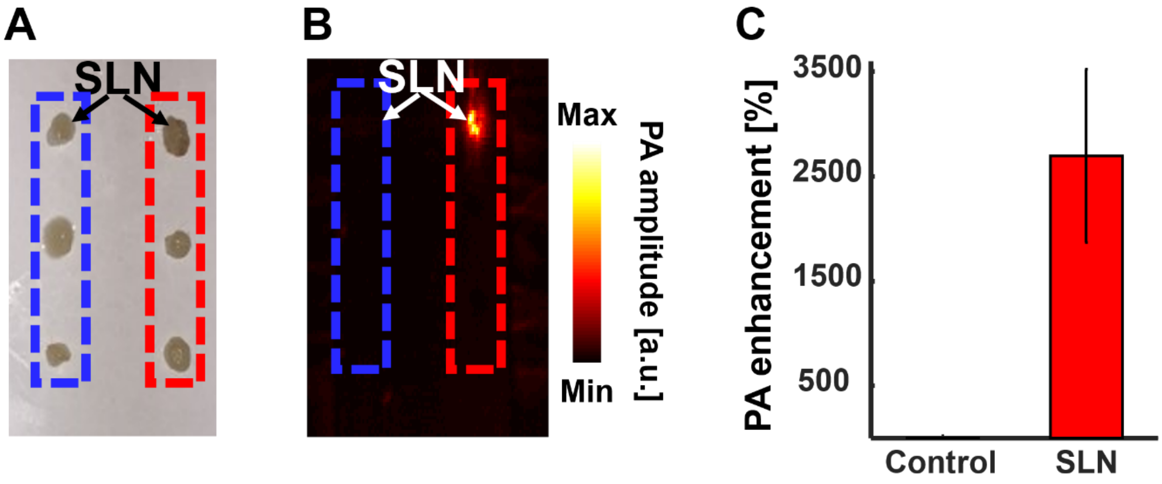

Figure S16. In vivo PA imaging of the rat SLN using a 800 nm laser excitation. (A) Before, (B) after without chicken tissue and (C) after with chicken tissue injection of NiPNP PA MAP images. (D) PA enhancement comparison before injection of NiPNP, after injection of NiPNP without chicken tissue and after injection of NiPNP with stacking the chicken tissue. PA, photoacoustic; US, ultrasound; NiPNP, Ni(II) complex-containing polymeric nanoparticles; MAP, maximum amplitude projection; SLN, sentinel lymph node; H, head; T, tail; CT, chicken tissue.

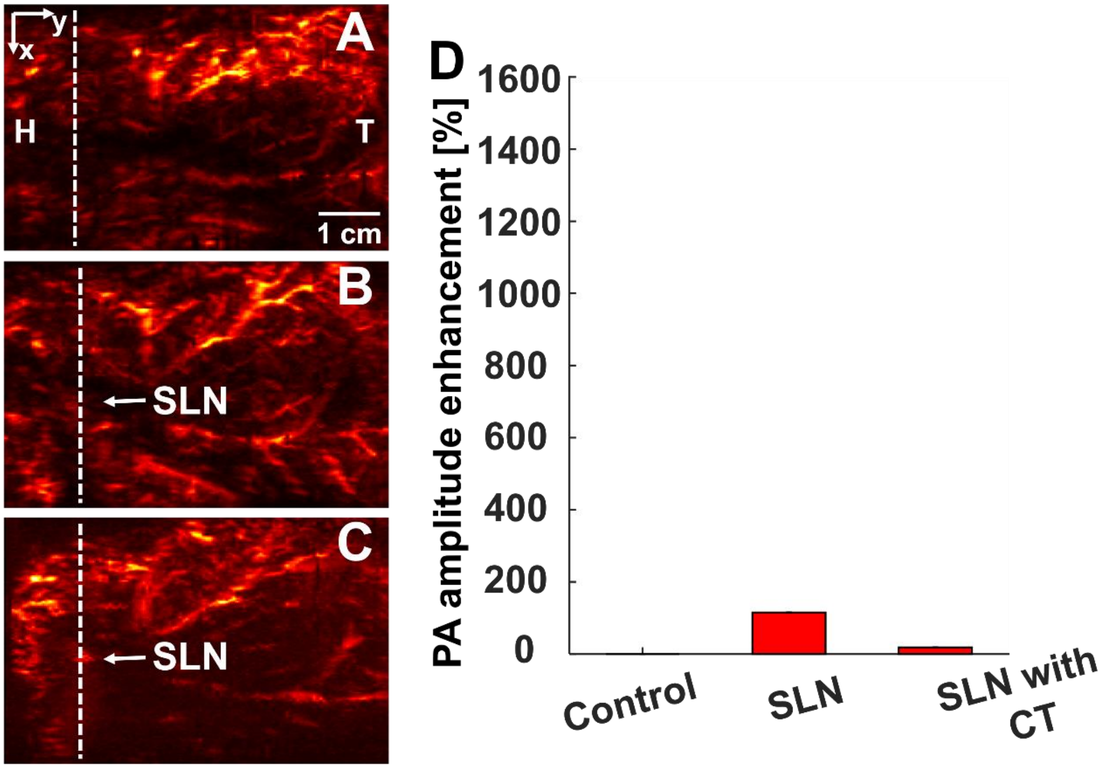

Figure S17. In vivo PA imaging of the NiPNP injected rat bladder using a 1064 nm laser excitation. (A) Overlaid PA/US image of bladder in the rat with stacking the chicken tissues. (B) PA enhancement comparison before injection of NiPNP and after injection of NiPNP with stacking the chicken tissues. PA, photoacoustic; US, ultrasound; NiPNP, Ni(II) complex-containing polymeric nanoparticles; CT, chicken tissue.

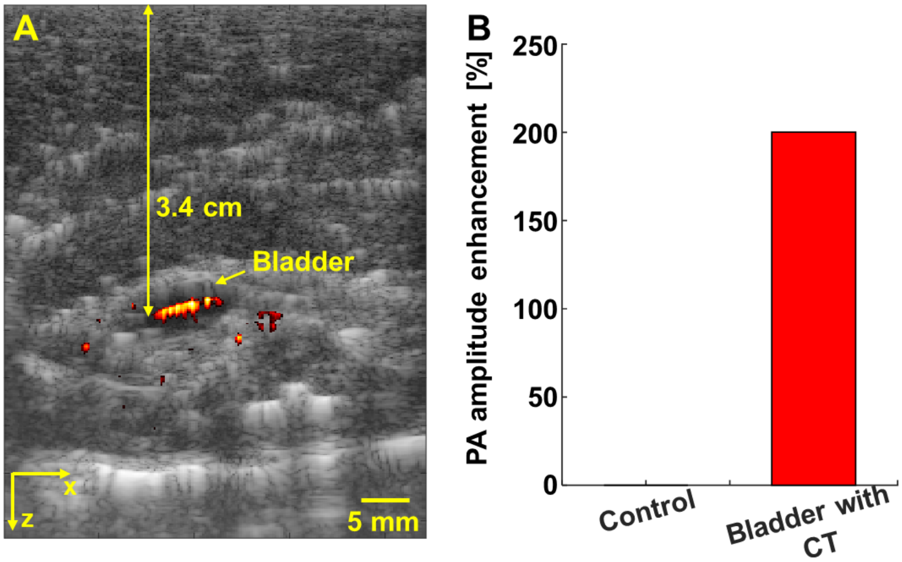

Table S1. Second near-infrared photoacoustic agents. TR, transducer; PAT, photoacoustic tomography; PACT, photoacoustic computed tomography and N/A, not available.

Ref.	Contrast agent						Imaging system		Experiments		
	Material	Confirmed biocompatibility	Overall size (nm)	Concentration in vitro; in vivo	Peak absorption wavelength (nm)	Photostability (shots)	Type	Laser power (mJ/cm^2) in vitro; in vivo	Imaging application	In vitro maximum depth (cm)	In vivo maximum depth (cm)
[37]	Copper sulfide nanoparticles	Cell viability	11	96 μg/mL	990	N/A	Single TR PAT	N/A	Penetration, brain cortex, lymph node	5	~mm
[39]	Phosphorus phthalocyanine	Biodistribution	N/A	25 mg/mL	997	N/A	Clinical PAT	56	Penetration, tumor	11.6	~mm
[40]	Semiconducting polymer nanoparticles	Cell viability	50-60	1 mg/mL; 6 mg/mL	1253	N/A	Single TR PAT	20; 5.5	Penetration, brain cortex	4	~mm
[41]	Semiconducting polymer nanoparticles	Cell viability	80-90	40 μg/mL	1210	12000	Clinical PAT	55; 46	Penetration, tumor	5.3	~mm
[42]	Bi_2Se_3 Nanoplates	N/A	72	15.3 mg/mL	700-850	N/A	Clinical PAT	76	Penetration, lymph node, cystography, GI tract	4.6	1.25
[43]	Polymer nanoparticles	Cell viability, biodistribution	171	1.94 mg/kg	1050-1150	N/A	Clinical PAT	N/A	Tumor	N/A	~mm
[44]	Gold nanorods	N/A	49 x 8	N/A	1000-1100	200	Clinical PAT	25	Tumor target	N/A	~mm
[47]	Semiconducting polymer nanoparticles	Cell viability, biodistribution	30	0.25-1.1 mg/mL	1079	N/A	Single TR PAT	N/A	Brain, tumor	N/A	~mm
[49]	Mesoporous silica nanoparticles	Cell viability, biodistribution	145	1 mg/mL; 2 mg/mL	900	N/A	Single TR PAT	100; 5.5	Penetration, tumor target	2	~mm
[50]	Charge-transfer nanocomplex	Cell viability	<100	1 mg/mL	750-1200	N/A	Single TR PAT	20; 5.5	Penetration, tumor	5	~mm
[51]	Semiconducting polymer nanoparticles	Cell viability, biodistribution	113	0.57 mg/mL; 50 mg/mL	1025	Confirmed	Clinical PAT	5; 10	Penetration, tumor	1.5	~mm
[52]	Surfactant-stripped micelles	Cell viability, biodistribution	30	15 mg/mL; 60 mg/kg	1040-1120	N/A	Clinical PAT	45; 62	Penetration, lymph node, tumor	12	3.1
This work	Nickel(II) Dithioloene-Containing Polymeric Nanoparticles	Cell viability, biodistribution	130	17.3 mg/mL	1064	3000	Clinical PAT	40; 66	Penetration, lymph node, cystography, GI tract	5.1	3.4
